# In-hospital course of children with COVID-19 infection - Results of the German nationwide inpatient sample

**DOI:** 10.1016/j.puhip.2025.100638

**Published:** 2025-07-01

**Authors:** Karsten Keller, Ingo Sagoschen, Volker H. Schmitt, Stefano Barco, Visvakanth Sivanathan, Omar Hahad, Frank P. Schmidt, Christine Espinola-Klein, Stavros Konstantinides, Thomas Münzel, Lukas Hobohm

**Affiliations:** a Department of Cardiology, University Medical Center of the Johannes Gutenberg-University Mainz, Mainz, Germany; b Center for Thrombosis and Hemostasis (CTH), University Medical Center of the Johannes Gutenberg-University Mainz, Mainz, Germany; c Medical Clinic VII, Department of Sports Medicine, University Hospital Heidelberg, Heidelberg, Germany; d German Center for Cardiovascular Research (DZHK), Partner Site Rhine Main, Mainz, Germany; e Department of Angiology, University Hospital Zurich, Zurich, Switzerland; f Department of Gastroenterology, University Medical Center Mainz (Johannes Gutenberg-University Mainz), Mainz, Germany; g Department of Cardiology, Mutterhaus Trier, Germany; h Department of Cardiology, Democritus University of Thrace, Alexandroupolis, Greece

**Keywords:** COVID-19, Case-fatality, Mortality, Ventilation, Childhood

## Abstract

**Objectives:**

To date, few large studies of clinical outcomes in pediatric COVID-19 patients have been reported.

**Study design:**

Epidemiological study of the German nationwide inpatient study (GNIS).

**Methods:**

We used the GNIS to analyze all hospitalized children ≤18 years with confirmed COVID-19 diagnosis in Germany between Jan 1st and December 31st, 2020.

**Results:**

Overall, 3360 children aged ≤18 years were hospitalized with COVID-19 infection in Germany in 2020 (49.8 % females). Among these, 1640 (48.8 %) were aged ≤6 years, 504 (15.0 %) 7 - ≤12 years and 1216 (36.2 %) were aged 13 - ≤18 years. Among these 3360 patients, 3.3 % were treated with mechanical ventilation and 0.23 % died in the hospital. The frequency of venous thromboembolism (0.18 %), vasculopathy (0.68 %), multisystem inflammatory syndrome caused by COVID-19 (0.65 %), and diagnosis of myocarditis (0.60 %) were low. Besides pneumonia and acute respiratory distress syndrome, obesity (OR 6.1 [95 %CI 2.1–18.2], P = 0.001), heart failure (OR 17.0 [95 %CI 6.8–42.1], P < 0.001) and acute/chronic kidney failure (OR 9.5 [95 %CI 4.0–22.2], P < 0.001) were independently associated with mechanical ventilation. Acute or chronic kidney failure (OR 41.4 [95 %CI 7.8–218.6], P < 0.001), liver disease (OR 18.8 [95 %CI 2.5–143.3], P = 0.005), and necessity of mechanical ventilation (OR 7.6 [95 %CI 1.2–47.4], P = 0.031) were independent risk factors for case-fatality.

**Conclusions:**

In Germany in 2020, hospitalized children aged ≤18 years with COVID-19 infection had a low case-fatality. Heart, liver and renal failure were associated with adverse COVID-19 complications, such as the need for mechanical ventilation or death. Myocarditis, vasculopathy and venous thromboembolism were rare complications in this patient group.

## 1 Introduction

First patient-cases of pneumonia caused by severe acute respiratory syndrome coronavirus type 2 (SARS-CoV-2) were detected in China in early December 2019 [[1], [2], [3], [4]]. Since these initial reports of a cluster of pneumonia cases, the coronavirus disease 2019 (COVID-19) has spread throughout the world, igniting the 21st century's deadliest pandemic [5]. The first infection-cases in Germany were reported at the end of January 2020 [6]. Sustained transmission of SARS-CoV-2 resulted in a strong spread of COVID-19 in the German population [3,4,[7], [8], [9]].

Most children and adolescents experience mild COVID-19 after SARS-CoV-2-infection in comparison to adults [[10], [11], [12]]. Asymptomatic infection is common in this young age-group [10,12]. The COVID-19 infection in children is usually of short duration and is accompanied by a low symptom burden. Nevertheless, some infected children revealed prolonged illness duration [12]. Although SARS-CoV-2 infection is common in children, data about the outcome and risk factors of adverse outcomes in this age group are limited [10,[13], [14], [15], [16]]. In this context, a holistic approach for children with persistent illness during the pandemic has to be established and risk factors of adverse outcomes have to be identified [10,12]. This is of outstanding interest to understand the determinants of adverse outcomes and the necessity of expansion of treatment requirements, which are both crucial factors for adequate healthcare planning, decision making, and pandemic management [3,4,17]. However, it should be noted that the evidence in this research context is limited and that the preferred sources tend to come from wealthy OECD countries [15,[18], [19], [20], [21], [22], [23], [24], [25], [26], [27], [28], [29]]. Although our analysis focuses on the health research of Germany, it must be taken into account that, with regard to the pandemic, a global view/perspective must not be forgotten and that poorer countries, and especially those in Africa and South America, should also be taken into account [[29], [30], [31], [32], [33], [34]].

Thus, the objective of the present study was to investigate the time trends of COVID-19 infection in hospitalized children and young patients in a large German nationwide inpatient sample in 2020 and to identify risk factors for adverse in-hospital outcomes in non-vaccinated children and young patients.

## 2 Methods

### 2.1 Data source

Statistical analyses of the present study were conducted on our behalf by the Research Data Center (RDC) of the Federal Bureau of Statistics (Wiesbaden, Germany). The aggregated statistics were provided by the RDC based on the generated and supplied SPSS codes (IBM Corp. Released 2011. IBM SPSS Statistics for Windows, Version 20.0. IBM Corp: Armonk, NY, USA). Therefore, this study analyzes a nationwide inpatient sample (NIS) from Germany (source: RDC of the Federal Statistical Office and the Statistical Offices of the federal states, DRG Statistics 2020, own calculations) [3,35].

With this data analysis of the German NIS, we aimed to analyze temporal trends of all hospitalized children (aged ≤18 years) with a confirmed COVID-19 diagnosis (ICD-code U07.1) during the observation period between January 1st and December 31, 2020, and to identify independent predictors of adverse in-hospital outcomes.

In addition, we performed a monthly trend analysis of liver diseases (ICD codes B15-B19 and K70-K77), renal diseases (N17-N19), and heart failure (I50) in all hospitalized children (aged ≤18 years). For this purpose, we asked the RDC to analyze the total monthly number of children (aged ≤18 years) using ICD codes. Of these aggregated data, the differences between 2020 and the reference year 2019 were calculated (Statistisches Bundesamt, DEStatis, source: DRG-Statistik, Sonderauswertung des Statistischen Bundesamtes). We decided to choose this rather unconventional definition for children aged ≤18 years, since we took our cues from other publications [36,37].

### Ethical statement

Since our present conducted study did not comprise a direct access by us as the investigators to individual patient data but only an access to summarized results provided by the RDC, an approval of an ethics panel or Institutional Review Board as well as patients’ informed consent were not required, in accordance with German law [3,35].

### 2.2 Coding of diagnoses, surgeries, procedures and definitions

In Germany, diagnosis- and procedure-related remuneration were first introduced in 2004. According to the German Diagnosis Related Groups (G-DRG) system, patient data on diagnoses, coexisting conditions, and surgeries as well as on procedures/interventions in combination with transferring these codes to the Institute for the Hospital Remuneration System is mandatory for German hospitals to receive their remuneration [3,35]. In this context, patient diagnoses are coded according to the International Statistical Classification of Diseases and Related Health Problems, 10th revision, with German modification (ICD-10-GM) [3,35]. Additionally, surgical, diagnostic, and interventional procedures were coded with OPS codes (Operationen-und Prozedurenschlüssel). In this analysis of the German NIS, we included all hospitalized patients aged ≤18 years with a confirmed COVID-19 diagnosis (ICD-code U07.1) in Germany during the entire year of 2020 (COVID-19 as main or secondary diagnosis).

Recurrent COVID infection was defined as the status of previously survived COVID-19 infection before the patient's hospitalization with the recurrent COVID-19 infection.

### 2.3 Statistical analysis

All hospitalized COVID-19 patients aged ≤18 years in Germany were included in this analysis and stratified in three groups of 6-year cycles: children aged ≤6 years were compared to those aged 7 to ≤12 years and to children aged 13 to ≤18 years.

Differences in patient characteristics between the groups of hospitalized COVID-19-patients of the three age-groups were statistically tested with Kruskal-Wallis-test for continuous variables and Fisher's exact or chi [2] test for categorical variables, as appropriate. Temporal trends regarding hospitalizations of COVID-19-patients, necessity of mechanical ventilation and in-hospital mortality over time and with age were estimated by means of linear regression analyses. The computed results are presented as beta (β)-estimates with corresponding 95 % confidence intervals (CI).

We calculated logistic regression models to investigate i) associations between patients’ characteristics, cardiovascular risk factors and comorbidities as well as manifestations of COVID-19, adverse events on the one hand and necessity of mechanical ventilation or case-fatality on the other hand. Results were presented as Odds Ratios (OR) and 95 % confidence intervals (CI). The multivariate regression models were adjusted for one of the two following models (with all of the variables in one model):

• Adjusted for sex
• Adjusted for age, sex, heart failure, acute and chronic lung diseases (including bronchial asthma, bronchitis, chronic obstructive pulmonary disease, emphysema, and/or bronchiectasis), and acute and/or chronic kidney failure.

We selected this epidemiological approach for adjustment to guarantee a widespread independence of these associations regarding the parameters included in the adjustment, which are well-known drivers of aggravated outcome during hospitalization.

All statistical analyses were calculated with the SPSS software (IBM Corp. Re-leased 2011. IBM SPSS Statistics for Windows, Version 20.0. IBM Corp: Armonk, NY, USA). Only P values of <0.05 (two-sided) were considered as statistically significant. No adjustment for multiple testing was applied.

## 3 Results

### 3.1 Baseline characteristics

Overall, 3360 cases of hospitalized patients aged ≤18 years with confirmed COVID-19 infection were diagnosed in Germany in 2020 (49.8 % females). The median age of the admitted children with COVID-19 was 7.0 years (IQR 0.0–15.0) with a median length of in-hospital stay of 2.0 days (IQR 1.0–4.0).

When stratified for 6-year periods, 1640 (48.8 %) patients were aged ≤6 years, 504 (15.0 %) were aged 7–12 years, and 1216 (36.2 %) were aged 13–18 years (Table 1).

**Table 1:** Patients’ characteristics, medical history, presentation and adverse in-hospital events of the 3360 hospitalized children with confirmed COVID-19 infection in Germany in the year 2020 stratified for 6-year cycle.

Parameters	Children with COVID-19 aged ≤6 years (n = 1640; 48.8 %)	Children with COVID-19 aged 7 to ≤12 years (n = 504; 15.0 %)	Children with COVID-19 aged 13 to ≤18 years (n = 1216; 36.2 %)	P-value
**Female sex**	709 (43.2 %)	214 (42.5 %)	751 (61.8 %)	**<0.001**
**Median length of in-hospital stay (days)**	2.0 (1.0–4.0)	2.0 (1.0–4.0)	2.0 (1.0–4.0)	**0.048**
**Cardiovascular risk factors**				
**Obesity**	1 (0.1 %)	8 (1.6 %)	33 (2.7 %)	**<0.001**
**Diabetes mellitus**	5 (0.3 %)	10 (2.0 %)	32 (2.6 %)	**<0.001**
**Essential arterial hypertension**	3 (0.2 %)	1 (0.2 %)	14 (1.2 %)	**0.001**
**Hyperlipidaemia**	1 (0.1 %)	0 (0 %)	1 (0.1 %)	0.816
**Comorbidities**				
**Coronary artery disease**	2 (0.1 %)	1 (0.2 %)	0 (0 %)	0.376
**Heart failure**	12 (0.7 %)	4 (0.8 %)	9 (0.7 %)	0.990
**Atrial fibrillation/flutter**	0 (0 %)	0 (0 %)	3 (0.2 %)	0.071
**Acute and chronic lung diseases (including bronchial asthma, bronchitis, chronic obstructive pulmonary disease, emphysema, and/or bronchiectasis)**	4 (0.2 %)	13 (2.6 %)	50 (4.1 %)	**<0.001**
**Bronchial asthma**	3 (0.2 %)	11 (2.2 %)	45 (3.7 %)	**<0.001**
**Chronic obstructive pulmonary disease**	0 (0 %)	0 (0 %)	2 (0.2 %)	0.171
**Acute and/or chronic kidney failure**	11 (0.7 %)	7 (1.4 %)	23 (1.9 %)	**0.012**
**Chronic renal insufficiency (glomerular filtration rate <60 ml/min/1,73 m^2^)**	3 (0.2 %)	2 (0.4 %)	9 (0.7 %)	0.073
**Cancer**	29 (1.8 %)	17 (3.4 %)	18 (1.5 %)	**0.028**
**Liver disease**	6 (0.4 %)	8 (1.6 %)	17 (1.4 %)	**0.004**
**Severe liver disease**	5 (0.3 %)	7 (1.4 %)	10 (0.8 %)	**0.020**
**Manifestations of COVID-19**				
**Acute bronchitis**	40 (2.4 %)	7 (1.4 %)	35 (2.9 %)	0.190
**Pneumonia**	69 (4.2 %)	25 (5.0 %)	120 (9.9 %)	**<0.001**
**Acute respiratory distress syndrome**	4 (0.2 %)	3 (0.6 %)	6 (0.6 %)	0.407
**Vasculopathy**	14 (0.9 %)	5 (1.0 %)	4 (0.3 %)	0.161
**Multisystem Inflammatory syndrome caused by COVID-19**	11 (0.7 %)	3 (0.6 %)	8 (0.7 %)	0.983
**Recurrent COVID-19 infection after previous COVID-19-infection**	4 (0.2 %)	0 (0 %)	3 (0.2 %)	0.538
**Treatment**				
**Mechanical ventilation**	69 (4.2 %)	15 (3.0 %)	26 (2.1 %)	**0.008**
**Extracorporeal membrane oxygenation (ECMO)**	1 (0.1 %)	2 (0.4 %)	1 (0.1 %)	0.144
**Dialysis**	0 (0 %)	1 (0.2 %)	6 (0.5 %)	**0.017**
**Adverse events during hospitalization**				
**In-hospital case-fatality**	2 (0.1 %)	3 (0.6 %)	3 (0.2 %)	0.162
**Cardio-pulmonary resuscitation**	2 (0.1 %)	1 (0.2 %)	1 (0.1 %)	0.816
**Venous thromboembolism**	3 (0.2 %)	0 (0 %)	3 (0.2 %)	0.543
**Pulmonary embolism**	0 (0 %)	0 (0 %)	1 (0.1 %)	0.414
**Acute kidney failure**	5 (0.3 %)	5 (1.0 %)	11 (0.9 %)	0.070
**Myocarditis**	5 (0.3 %)	4 (0.8 %)	11 (0.9 %)	0.098
**Myocardial infarction**	0 (0 %)	0 (0 %)	0 (0 %)	1.000
**Stroke (ischaemic or haemorrhagic)**	0 (0 %)	1 (0.2 %)	2 (0.2 %)	0.234
**Intracerebral bleeding**	0 (0 %)	0 (0 %)	0 (0 %)	1.000
**Gastro-intestinal bleeding**	8 (0.5 %)	5 (1.0 %)	7 (0.6 %)	0.434
**Transfusion of blood constituents**	35 (2.1 %)	19 (3.8 %)	22 (1.8 %)	**0.040**

While the majority of children admitted to German hospitals in the first two 6-year cycles were male, hospitalized children aged 13–18 years were more often female. Cardiovascular risk factors were rare in all the investigated 6-year-cycles of these children; nevertheless, we detected a small increase in diabetes mellitus, obesity, and arterial hypertension in the older 6-year cycles (Table 1).

Although the prevalence of cardiovascular comorbidities was similar between the groups, they differed in terms of cancer and liver disease. Regarding the manifestations of COVID-19, the proportion of pneumonia approximately doubled in the oldest 6-year-cycle, while all other respiratory manifestations were comparable between the groups (Table 1). We analyzed the social need for admission of COVD-19-infected children: In the patients aged ≤6 years, a social need for admission was coded in 675 hospitalizations (41.2 %), while this proportion decreased to 14.5 % (n = 73) children aged 7–12 years and 1.9 % (n = 23) in hospitalized children aged 13–18 years.

The greatest need for mechanical ventilation was observed in the youngest patients aged ≤6 years (Table 1).

Fortunately, the frequency of adverse in-hospital outcomes was low. Eight children aged ≤18 years died during their in-hospital stay in Germany in 2020 (0.23 %). If we exclude hospitalized children with a social need for hospital admission, the in-hospital case fatality rate would be the highest, computed at 0.31 %. The highest fatality rate was observed in children aged 7–12 years (0.6 %). Venous thromboembolism (VTE) occurred in six children (0.18 %), and vasculopathy (0.68 %), multisystem inflammatory syndrome caused by COVID-19 (0.65 %), and myocarditis were more prevalent (0.60 %) with increasing age. The need for transfusion of blood constituents was highest in the middle 6-year cycle of children aged 7–12 years, accounting for 3.8 % of all admitted patients (Table 1).

### 3.2 Monthly and age-dependent trends

**Fig. 1:**
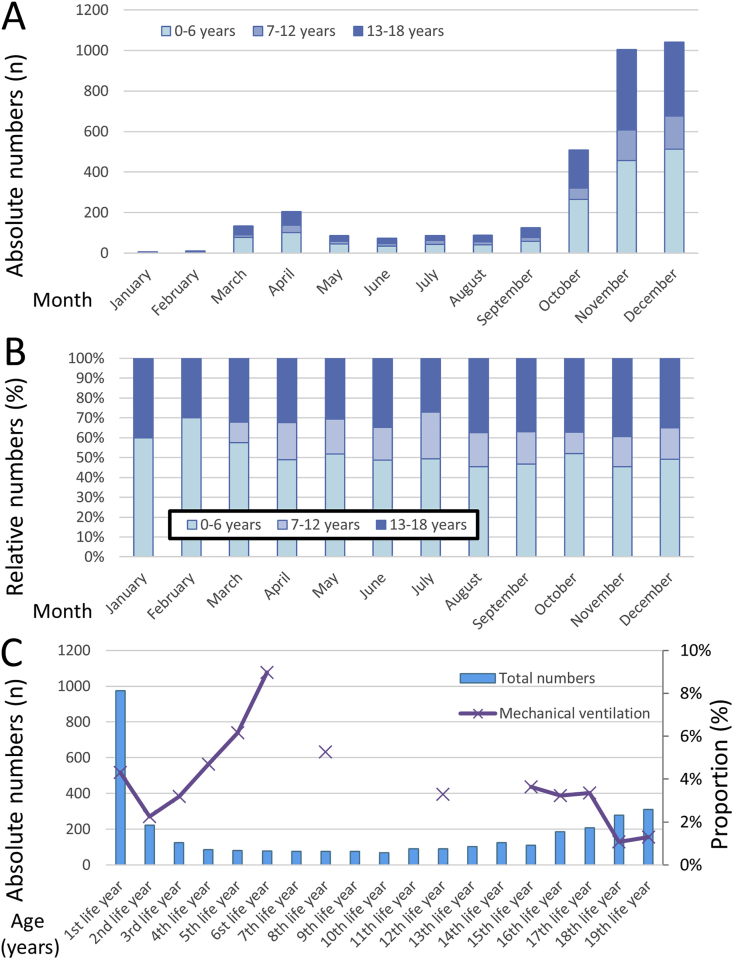
Temporal trends regarding total numbers of hospitalized childhood patients with COVID-19-infection stratified by age classes **Panel A –** Temporal trends regarding absolute numbers of hospitalized childhood patients with COVID-19-infection stratified by age classes **Panel B –** Temporal trends regarding relative numbers of hospitalized childhood patients with COVID-19-infection stratified by age classes **Panel C –** Temporal trends regarding absolute numbers of hospitalized childhood patients with COVID-19-infection stratified by age and proportion regarding necessity of mechanical ventilation (dark blue line).

The highest monthly admission numbers of hospitalized children and young people aged ≤18 years were observed for October, November, and December 2020 (Fig. 1A). While the highest proportion of children aged ≤6 years was detected in the winter months, the highest percentage in children aged 7–12 years was seen in summer, and that of children aged 13–18 years was noticed for the months of January, August, and September (Fig. 1B). We observed the highest need for mechanical ventilation in the 1st, and 4th to 8th life-year; statistically, the risk that children have to be ventilated decreased with increasing age (β −2.2 [95 %CI -3.5 to −0.8], P = 0.002) (Fig. 1C).

### 3.3 Regional trends

**Fig. 2:**
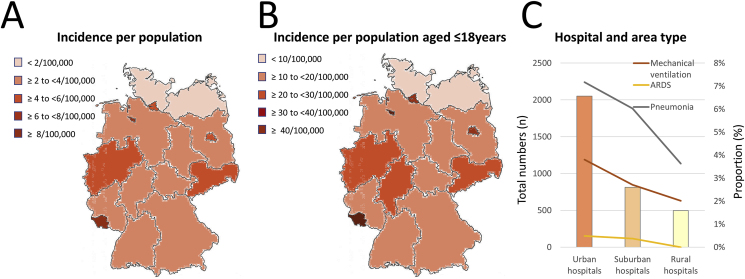
Regional differences regarding total numbers, incidence and outcomes of hospitalized childhood patients with COVID-19-infection **Panel A –** Incidence of hospitalized childhood patients with COVID-19-infection related to the general population **Panel B –** Incidence of hospitalized childhood patients with COVID-19-infection related to the population aged ≤18 years **Panel C –** Regional trends regarding rates of pneumonia, ARDS and mechanical ventilation of hospitalized childhood patients with COVID-19-infection (comparison of hospitals in urban vs. suburban vs. rural areas).

We detected the lowest incidence for hospitalized children and young people aged ≤18 years with confirmed COVID-19 infection in northern Germany (Federal States of Schleswig-Holstein and Mecklenburg-Vorpommern), whereas the highest incidence was found in the federal state of Saarland, as well as in the city-states of Berlin, Bremen, and Hamburg (Fig. 2A and B). The largest number of hospitalized children and young people aged ≤18 years with confirmed COVID-19 infection were treated in hospitals in the urban areas of Germany, with the highest burden of pneumonia and ARDS cases (Fig. 2C). Consequently, the highest proportion of patients who required mechanical ventilation was identified in urban hospitals (urban, 3.8 %; suburban, 2.7 %; and rural, 2.0 %). Only in two children, who were treated in urban hospitals, an ECMO was initiated. While none of the hospitalized children and young people aged ≤18 years with confirmed COVID-19 infection treated in rural hospitals died during the observational period, eight patients treated in suburban and urban hospitals died in 2020.

### 3.4 Comparison of patients with necessity of mechanical ventilation VS. Those without mechanical ventilation

Overall, 110 children and young people aged ≤18 years with confirmed COVID-19 (3.3 %) were treated with mechanical ventilation (Table S1 of the supplementary material). Notably, patients with mechanical ventilation were more common of male sex (60.0 % vs. 49.8 %, P = 0.036), had pneumonia more often (33.6 % vs. 5.5 %, P < 0.001), ARDS (6.4 % vs. 0.2 %, P < 0.001), heart failure (10.0 % vs. 0.4 %, P < 0.001), acute or chronic kidney failure (10.0 % vs. 0.9 %, P < 0.001), and myocarditis (3.6 % vs. 0.5 %, P = 0.004) (Table S1 in the supplementary material).

## 4 Comparison of survivors VS. NON-SURVIVORS

As mentioned previously, only eight hospitalized children and young people aged ≤18 years with confirmed COVID-19 infection (0.2 %) died during the in-hospital course (Table 2). Non-survivors had a higher proportion of acute and chronic kidney failure (37.5 % vs. 1.1 %, P < 0.001), whereas cardiovascular disease was not more prevalent in any group. Unsurprisingly, the deceased patients had a higher need for mechanical ventilation (Table 2).

**Table 2:** Patients’ characteristics, medical history, presentation and adverse in-hospital events of the 3360 hospitalized children with confirmed COVID-19 infection in Germany in the year 2020 stratified for survival.

Parameters	All hospitalized children with COVID-19 aged ≤18 years (n = 3360; 100.0 %)	Children with COVID-19, who were discharged alive (n = 3352; 99.8 %)	Children with COVID-19, who died during hospitalization (n = 8; 0.2 %)	P-value
**Age (years)**	7.0 (0.0–15.0)	7.0 (0.0–15.0)	8.0 (4.0–14.8)	0.443
**Female sex**	1674 (49.8 %)	1671 (49.9 %)	3 (37.5 %)	0.726
**Length of in-hospital stay (days)**	2.0 (1.0–4.0)	2.0 (1.0–4.0)	15.0 (1.6–34.0)	**0.020**
**Cardiovascular risk factors**				
**Obesity**	42 (1.3 %)	42 (1.3 %)	0 (0.0 %)	0.750
**Essential arterial hypertension**	18 (0.5 %)	18 (0.5 %)	0 (0.0 %)	0.835
**Comorbidities**				
**Coronary artery disease**	3 (0.1 %)	3 (0.1 %)	0 (0 %)	1.000
**Heart failure**	25 (0.7 %)	a	a	0.058
**Atrial fibrillation/flutter**	3 (0.1 %)	3 (0.1 %)	0 (0.0 %)	1.000
**Acute and chronic lung diseases (including bronchial asthma, bronchitis, chronic obstructive pulmonary disease, emphysema, and/or bronchiectasis)**	67 (2.0 %)	67 (2.0 %)	0 (0.0 %)	1.000
**Bronchial asthma**	59 (1.8 %)	59 (1.8 %)	0 (0.0 %)	1.000
**Acute and/or chronic kidney failure**	41 (1.2 %)	38 (1.1 %)	3 (37.5 %)	**<0.001**
**Cancer**	64 (1.9 %)	a	a	0.143
**Hepatitis**	3 (0.1 %)	3 (0.1 %)	0 (0.0 %)	1.000
**Liver disease**	31 (0.9 %)	a	a	**0.002**
**Severe liver disease**	22 (0.7 %)	a	a	**0.001**
**Manifestations of COVID-19**				
**Acute bronchitis**	82 (2.4 %)	82 (2.4 %)	0 (0.0 %)	1.000
**Pneumonia**	214 (6.4 %)	a	a	**<0.001**
**Acute respiratory distress syndrome**	13 (0.4 %)	a	a	**<0.001**
**Vasculopathy**	23 (0.7 %)	23 (0.7 %)	0 (0.0 %)	1.000
**Multisystem Inflammatory syndrome caused by COVID-19**	22 (0.7 %)	22 (0.7 %)	0 (0.0 %)	1.000
**Recurrent COVID-19 infection after previous COVID-19-infection**	7 (0.2 %)	7 (0.2 %)	0 (0.0 %)	1.000
**Treatment**				
**Mechanical ventilation**	110 (3.3 %)	107 (3.2 %)	3 (37.5 %)	**0.002**
**Adverse events during hospitalization**				
**Venous thromboembolism**	6 (0.2 %)	6 (0.2 %)	0 (0.0 %)	1.000
**Acute kidney failure**	21 (0.6 %)	18 (0.5 %)	3 (37.5 %)	**<0.001**
**Myocardial infarction**	0 (0 %)	0 (0 %)	0 (0 %)	1.000
**Stroke (ischaemic or haemorrhagic)**	3 (0.1 %)	a	a	**0.007**
**Intracerebral bleeding**	0 (0 %)	0 (0 %)	0 (0 %)	1.000
**Transfusion of blood constituents**	76 (2.3 %)	71 (2.1 %)	5 (62.5 %)	**<0.001**

a due to confidentiality reasons, the data cannot be shown.

### 4.1 Independent predictors of mechanical ventilation

Strong and independent factors associated with mechanical ventilation in children and young people aged ≤18 years with confirmed COVID-19 infection were obesity (OR 6.1 [95 %CI 2.1–18.2], P = 0.001), arterial hypertension (OR 8.1 [95 %CI 2.2–30.1], P = 0.002), heart failure (OR 17.0 [95 %CI 6.8–42.1], P < 0.001), acute and chronic kidney failure (OR 9.5 [95 %CI 4.0–22.2], P < 0.001), VTE (OR 16.1 [95 %CI 2.2–119.5], P = 0.007), and the acute manifestation entities of COVID-19 comprising pneumonia (OR 9.4 [95 %CI 5.9–15.2], P < 0.001), and ARDS (OR 23.4 [95 %CI 6.1–90.5], P < 0.001) (Table S2 of the supplementary material).

While pneumonia and ARDS were important independent factors regarding the necessity of mechanical ventilation in all three 6-year cycles, liver diseases and VTE were associated with mechanical ventilation in the first 6-year cycle only, whereas acute and chronic kidney failure were associated with mechanical ventilation in the first two 6-year cycles, heart failure in the first and third 6-year cycles, cancer in the second, and obesity in the oldest 6-year cycle (Table S3 of the supplementary material).

### 4.2 Independent predictors OF IN-HOSPITAL case-fatality

We identified pneumonia (OR 33.1 [95 %CI 5.8–188.1], P < 0.001), ARDS (OR 24.1 [95 %CI 3.1–189.5], P = 0.002), acute and chronic kidney failure (OR 41.4 [95 %CI 7.8–218.6], P < 0.001), liver disease (OR 18.8 [95 %CI 2.5–143.3], P = 0.005), and necessity of mechanical ventilation (OR 7.6 [95 %CI 1.2–47.4], P = 0.031) as strong independent predictors of case-fatality (Table 3).

**Table 3:** Impact of patient-characteristics and conditions on case-fatality of the 3360 hospitalized children with confirmed COVID-19 infection in Germany in the year 2020 (univariate and multivariate logistic regression model).

	Univariate regression		Multivariate regression^a^		Multivariate regression^b^	
	OR (95 % CI)	P-value	OR (95 % CI)	P-value	OR (95 % CI)	P-value
Age (years)	1.0 (0.9–1.1)	0.575	1.0 (0.9–1.1)	0.496	1.0 (0.9–1.1)	0.785
Female sex	0.6 (0.1–2.5)	0.490	0.6 (0.1–2.5)	0.490	0.6 (0.1–2.4)	0.446
**Cardiovascular risk factors and comorbidities**						
Obesity	–	–	–	–	–	–
Diabetes mellitus	10.3 (1.2–85.1)	**0.031**	10.2 (1.2–85.0)	**0.031**	7.0 (0.6–78.7)	0.114
Heart failure	19.8 (2.3–167.3)	**0.006**	19.3 (2.3–163.6)	**0.007**	2.8 (0.2–33.9)	0.408
Myocarditis	25.1 (2.9–213.7)	**0.003**	24.0 (2.8–206.0)	**0.004**	3.5 (0.2–56.6)	0.370
Bronchial asthma	–	**-**	–	**-**	–	–
Acute and/or chronic kidney failure	52.3 (12.1–226.8)	**<0.001**	53.8 (12.4–234.4)	**<0.001**	41.4 (7.8–218.6)	**<0.001**
Acute kidney failure	111.1 (24.7–500.3)	**<0.001**	114.1 (25.1–517.4)	**<0.001**	100.5 (17.1–589.9)	**<0.001**
Cancer	7.5 (0.9–61.5)	0.062	7.2 (0.9–59.6)	0.067	4.3 (0.4–43.6)	0.223
Liver disease	38.2 (7.4–197.2)	**<0.001**	37.0 (7.1–192.0)	**<0.001**	18.8 (2.5–143.3)	**0.005**
Venous thromboembolism	–	–	–	–	–	–
**Manifestations of COVID-19**						
Acute bronchitis	–	–	–	–	–	–
Pneumonia	45.3 (9.1–226.1)	**<0.001**	44.8 (9.0–223.4)	**<0.001**	33.1 (5.8–188.1)	**<0.001**
Acute respiratory distress syndrome	101.2 (18.4–557.7)	**<0.001**	104.8 (18.8–583.8)	**<0.001**	24.1 (3.1–189.5)	**0.002**
Vasculopathy	–	–	–	–	–	–
Encephalopathy	–	–	–	–	–	–
Multi-segmental Inflammatory syndrome caused by COVID-19	–	–	–	–	–	–
**Treatment**						
Mechanical ventilation	18.2 (4.3–77.1)	**<0.001**	7.6 (1.2–47.4)	**<0.001**	7.6 (1.2–47.4)	**0.031**

a Adjusted for sex.

b Adjusted for age, sex, heart failure, acute and chronic lung diseases (including bronchial asthma, bronchits, chronic obstructive pulmonary disease, emphysema, and/or bronchiectasis), and acute and/or chronic kidney failure.

Pneumonia was associated with in-hospital case fatality in the first two 6-year cycles, whereas ARDS was the main risk factor in the second and third 6-year cycles (Table S4 of the supplementary material). Heart failure was associated with case fatality in the first 6-year cycle, whereas acute and chronic kidney failure were predictors of case fatality in all three 6-year cycles (Table S4 of the supplementary material).

However, the total number of liver and renal diseases as well as heart failure cases did not increase during the pandemic year 2020 in comparison to the pre-pandemic year 2019 in all hospitalized children and young people aged ≤18 years (Fig. S3 of the supplementary material).

### 4.3 Length OF IN-HOSPITAL stay

**Fig. 3:**
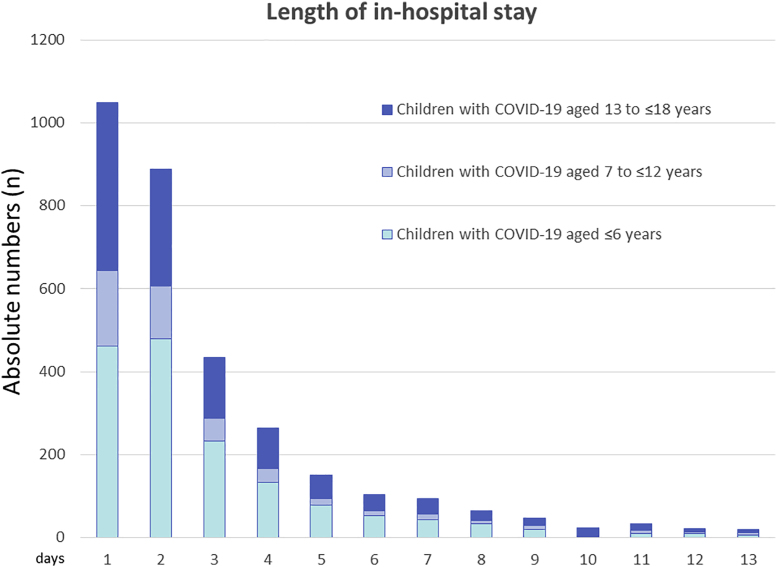
Length of in-hospital stay.

We detected no significant differences in the median in-hospital stay among the three age groups. In all age groups, the median length of in-hospital stay was 2.0 [1.0–4.0] days, respectively (Table 1). The total number of children with different lengths of in-hospital stay in different groups is shown in Fig. 3. Table 4 shows that heart, lung, kidney, and liver diseases as well as cancer and bleeding events triggered prolonged in-hospital stays.

**Table 4:** Patients’ characteristics, medical history, presentation and adverse in-hospital events of the 3360 hospitalized children with confirmed COVID-19 infection in Germany in the year 2020 stratified for prolonged length in-hospital stay >9 days.

Parameters	Children with COVID-19 hospitalized with a length in-hospital stay ≤9 days (n = 3095; 92.1 %)	Children with COVID-19 hospitalized with a length in-hospital stay >9 days (n = 265; 7.9 %)	P-value
**Age (years)**	7.0 (0.0–15.0)	9.0 (1.0–16.0)	**0.013**
**Female sex**	1554 (50.2 %)	120 (45.3 %)	0.124
**Cardiovascular risk factors**			
**Obesity**	37 (1.2 %)	5 (1.9 %)	0.331
**Essential arterial hypertension**	10 (0.3 %)	8 (3.0 %)	**<0.001**
**Diabetes mellitus**	32 (1.0 %)	15 (5.7 %)	**<0.001**
**Comorbidities**			
**Heart failure**	8 (0.3 %)	17 (6.4 %)	**<0.001**
**Acute and chronic lung diseases (including bronchial asthma, bronchits, chronic obstructive pulmonary disease, emphysema, and/or bronchiectasis)**	62 (2.0 %)	15 (5.7 %)	**<0.001**
**Bronchial asthma**	52 (1.7 %)	7 (2.6 %)	0.180
**Acute and/or chronic kidney failure**	24 (0.8 %)	17 (6.4 %)	**<0.001**
**Cancer**	45 (1.5 %)	19 (7.2 %)	**<0.001**
**Hepatitis**	3 (0.1 %)	0 (0.0 %)	0.782
**Liver disease**	21 (0.7 %)	10 (3.8 %)	**<0.001**
**Severe liver disease**	14 (0.5 %)	8 (3.0 %)	**<0.001**
**Manifestations of COVID-19**			
**Pneumonia**	153 (4.9 %)	61 (23.0 %)	**<0.001**
**Vasculopathy**	10 (0.3 %)	13 (4.9 %)	**<0.001**
**Multisystem Inflammatory syndrome caused by COVID-19**	11 (0.4 %)	11 (4.2 %)	**<0.001**
**Recurrent COVID-19 infection after previous COVID-19**	7 (0.2 %)	0 (0.0 %)	0.562
**Adverse events during hospitalization**			
**In-hospital case-fatality**	3 (0.1 %)	5 (1.9 %)	**<0.001**
**Acute kidney failure**	10 (0.3 %)	11 (4.2 %)	**<0.001**
**Myocarditis**	11 (0.4 %)	9 (3.4 %)	**<0.001**
**Myocardial infarction**	0 (0 %)	0 (0 %)	1.000
**Stroke (ischaemic or haemorrhagic)**	0 (0 %)	3 (1.1 %)	**<0.001**
**Intracerebral bleeding**	0 (0 %)	0 (0 %)	1.000
**Transfusion of blood constituents**	37 (1.2 %)	39 (14.7 %)	**<0.001**

### 4.4 Comparison of young children VS. Adolescents

Overall, 1861 (55.4 %) of the hospitalized COVID-19 patients aged ≤18 years were younger children <10 years and 1499 (44.6 %) were adolescents aged ≥10 to ≤18 years. While children <10 years were most often hospitalized in the first year of their life, adolescents were in median 16 years old (Table 5). Hospitalized adolescent COVID-19 patients were in the majority of hospitalization-cases female, while younger children were more often of male sex (58.2 % vs. 43.1 %, P < 0.001). As expected, all investigated cardiovascular risk factors were more prevalent in adolescent patients than in younger children hospitalized with COVID-19. Notably, adolescents were more often afflicted by acute and chronic lung (others than COVID-19), kidney and liver diseases (Table 5). Regarding COVID-19 manifestations, pneumonia (8.7 % vs. 4.5 %, P < 0.001) was more prevalent in adolescent patients, whereas vasculopathy was more often identified in younger children (1.0 % vs. 0.3 %, P = 0.034). All adverse in-hospital events including in-hospital case-fatality (P = 0.739) occurred similarly often in younger children and adolescent patients, whereby younger children were more often treated with mechanical ventilation than adolescent patients with COVID-19 (4.2 % vs. 2.1 %, P = 0.001) (Table 5).

**Table 5:** Patients’ characteristics, medical history, presentation and adverse in-hospital events of younger children (<10 years) and adolescents (≥10 to ≤18 years) with confirmed COVID-19 infection in Germany during the year 2020.

Parameters	Younger children (<10 years) (n = 1861; 55.4 %)	Adolescents (≥10 to ≤18 years) (n = 1499; 44.6 %)	P-value
**Age (years)**	0.0 (0.0–3.0)	16.0 (13.0–17.0)	**<0.001**
**Female sex**	802 (43.1 %)	872 (58.2 %)	**<0.001**
**Length of in-hospital stay (days)**	2.0 (1.0–4.0)	2.0 (1.0–4.0)	0.279
**Cardiovascular risk factors**			
**Obesity**	6 (0.3 %)	36 (2.4 %)	**<0.001**
**Essential arterial hypertension**	4 (0.2 %)	14 (0.9 %)	**0.007**
**Diabetes mellitus**	10 (0.5 %)	37 (2.5 %)	**<0.001**
**Comorbidities**			
**Coronary artery disease**	3 (0.2 %)	0 (0.0 %)	0.258
**Heart failure**	14 (0.8 %)	11 (0.7 %)	0.951
**Acute and chronic lung diseases (including bronchial asthma, bronchits, chronic obstructive pulmonary disease, emphysema, and/or bronchiectasis)**	16 (0.9 %)	61 (4.1 %)	**<0.001**
**Bronchial asthma**	7 (0.4 %)	52 (3.5 %)	**<0.001**
**Acute and/or chronic kidney failure**	16 (0.9 %)	25 (1.7 %)	**0.034**
**Cancer**	36 (1.9 %)	28 (1.9 %)	0.888
**Hepatitis**	0 (0.0 %)	3 (0.2 %)	0.089
**Liver disease**	11 (0.6 %)	20 (1.3 %)	**0.025**
**Severe liver disease**	10 (0.5 %)	12 (0.8 %)	0.393
**Manifestations of COVID-19**			
**Acute bronchitis**	45 (2.4 %)	37 (2.5 %)	0.925
**Pneumonia**	83 (4.5 %)	131 (8.7 %)	**<0.001**
**ARDS**	7 (0.4 %)	6 (0.4 %)	1.000
**Vasculopathy**	18 (1.0 %)	5 (0.3 %)	**0.034**
**Multisystem Inflammatory syndrome caused by COVID-19**	13 (0.7 %)	9 (0.6 %)	0.831
**Recurrent COVID-19 infection after previous COVID-19**	4 (0.2 %)	3 (0.2 %)	1.000
**Treatment**			
**Mechanical ventilation**	78 (4.2 %)	32 (2.1 %)	**0.001**
**Adverse events during hospitalization**			
**In-hospital case-fatality**	5 (0.3 %)	3 (0.2 %)	0.739
**Venous thromboembolism**	3 (0.2 %)	3 (0.2 %)	1.000
**Acute kidney failure**	9 (0.5 %)	12 (0.8 %)	0.276
**Myocarditis**	7 (0.4 %)	13 (0.9 %)	0.074
**Myocardial infarction**	0 (0 %)	0 (0 %)	1.000
**Stroke (ischaemic or haemorrha-gic)**	0 (0.0 %)	3 (0.2 %)	0.089
**Intracerebral bleeding**	0 (0 %)	0 (0 %)	1.000
**Gastro-intestinal bleeding**	12 (0.6 %)	8 (0.5 %)	0.823
**Transfusion of blood constituents**	46 (2.5 %)	30 (2.0 %)	0.362

### 4.5 Comparison of younger VS. Older adolescents

When focusing on the 1499 adolescent patients, only 408 patients were aged ≥10 to <14 years (27.2 %), whereas more than 2/3 of the adolescent patients (n = 1091; 72.8 %) were older (≥14 to ≤18 years) (Table 6). The majority of the older adolescents were female (62.1 %), while in younger adolescent patients more male patients were counted (52.2 %). The cardiovascular risk factors and the comorbidity profile were comparable between both groups (Table 6). Although pneumonia as one important manifestation of COVID-19 was more prevalent in older than in younger adolescent patients (10.4 % vs. 4.2 %, P < 0.001), both patients of both groups were similarly often treated with mechanical ventilation and in-hospital case-fatality rate was comparable (Table 6).

**Table 6:** Patients’ characteristics, medical history, presentation and adverse in-hospital events of younger (≥10 to <14 years) and older adolescents (≥14 to ≤18 years) with confirmed COVID-19 infection in Germany during the year 2020.

Parameters	Young adolescents (≥10 to <14 years) (n = 408; 27.2 %)	Older adolescents (≥14 to ≤18 years) (n = 1091; 72.8 %)	P-value
**Age (years)**	12.0 (11.0–13.0)	17.0 (15.0–18.0)	**<0.001**
**Female sex**	195 (47.8 %)	677 (62.1 %)	**<0.001**
**Length of in-hospital stay (days)**	2.0 (1.0–4.0)	2.0 (1.0–4.0)	**0.002**
**Cardiovascular risk factors**			
**Obesity**	6 (1.5 %)	30 (2.7 %)	0.185
**Essential arterial hypertension**	a	a	0.130
**Diabetes mellitus**	10 (2.5 %)	27 (2.5 %)	0.979
**Comorbidities**			
**Heart failure**	5 (1.2 %)	6 (0.5 %)	0.182
**Acute and chronic lung diseases (including bronchial asthma, bronchits, chronic obstructive pulmonary disease, emphysema, and/or bronchiectasis)**	16 (3.9 %)	45 (4.1 %)	0.859
**Bronchial asthma**	14 (3.4 %)	38 (3.5 %)	0.961
**Acute and/or chronic kidney failure**	6 (1.5 %)	19 (1.7 %)	0.824
**Cancer**	11 (2.7 %)	17 (1.6 %)	0.148
**Hepatitis**	a	a	1.000
**Liver disease**	4 (1.0 %)	16 (1.5 %)	0.616
**Severe liver disease**	3 (0.7 %)	9 (0.8 %)	1.000
**Manifestations of COVID-19**			
**Acute bronchitis**	5 (1.2 %)	32 (2.9 %)	0.062
**Pneumonia**	17 (4.2 %)	114 (10.4 %)	**<0.001**
**ARDS**	0 (0.0 %)	6 (0.5 %)	0.198
**Vasculopathy**	a	a	1.000
**Multisystem Inflammatory syndrome caused by COVID-19**	3 (0.7 %)	6 (0.5 %)	0.711
**Recurrent COVID-19 infection after previous COVID-19**	a	a	1.000
**Treatment**			
**Mechanical ventilation**	8 (2.0 %)	24 (2.2 %)	1.000
**Adverse events during hospitalization**			
**In-hospital case-fatality**	0 (0.0 %)	3 (0.3 %)	0.567
**Venous thromboembolism**	0 (0.0 %)	3 (0.3 %)	0.567
**Acute kidney failure**	4 (1.0 %)	8 (0.7 %)	0.745
**Myocarditis**	3 (0.7 %)	10 (0.9 %)	1.000
**Myocardial infarction**	0 (0 %)	0 (0 %)	1.000
**Stroke (ischaemic or haemorrha-gic)**	a	a	1.000
**Intracerebral bleeding**	0 (0 %)	0 (0 %)	1.000
**Gastro-intestinal bleeding**	3 (0.7 %)	5 (0.5 %)	0.455
**Transfusion of blood constituents**	13 (3.2 %)	17 (1.6 %)	**0.045**

a due to confidentiality reasons, the data cannot be shown.

## 5 Discussion

Studies have suggested that approximately 19 % of all COVID-19 cases occur in children [13]. Our study results support the findings of previous studies showing that children infected with SARS-CoV-2 usually suffer from a mild course of the disease and have a lower hospitalization rate than SARS-CoV-2-infected adults. However, data are scarce regarding the incidence and risk factors associated with adverse outcomes in this demographic [10,14,15,[38], [39], [40], [41]]. The primary objective of our study was to fill this gap using the German nationwide inpatients sample analyzing all hospitalized children and young people aged ≤18 years in Germany admitted to German hospitals during the year 2020 facing the original strain of SARS-CoV-2 and possibly also the alpha variant. Since the vaccination program against SARS-CoV-2 for adults started in most European countries, including Germany, in December 2020 and the approval of vaccines for children aged 12 years and older did not take place not before mid of 2021, the results of our study were not influenced by SARS-CoV-2 vaccination [4,42].

The main results of our study can be summarized as follows: I) The in-hospital case fatality rate was low at 0.23 % (n = 8 deaths). II) The highest fatality rate was observed in children aged 7–12 years (0.6 %). III) The frequency of mechanical ventilation was 3.3 % in children aged ≤6 years. IV) The prevalences of VTE (0.2 %) and myocarditis (0.6 %) were low. V) Obesity, heart failure, acute and chronic kidney failure, pneumonia, and ARDS are associated with mechanical ventilation. VII) The strong and independent predictors of case fatality were pneumonia, ARDS, acute and chronic kidney failure, liver disease, and the need for mechanical ventilation. VIII) We detected regional disparities in COVID-19 admissions in the German federal states.

Understanding the factors leading to poor outcomes and the need for expanded treatment options, especially in hospitalized pediatric patients, particularly in intensive care unit (ICU) and mechanical ventilation, is crucial for effective healthcare planning, decision-making, and pandemic management [3,4,17,22].

Similar to previously published age-unselected epidemiological studies, our results showed an increase in hospital admissions of children and young people with COVID-19 in the fall and winter of 2020 [3]. Although COVID-19-infected children suffer in the vast majority only from mild symptoms and also asymptomatic infections in this age group are common [10,12,15,22,39], the substantial increase of COVID-19 cases in the German general population during October, November, and December 2020 lead also in children and young people aged ≤18 years to a significant incline of hospitalizations. Our data also demonstrated a social need, especially for young COVID-19-infected children, which decreased with increasing age. Additionally, we identified regional disparities in the hospitalization rates of children with COVID-19. These differences in hospitalization rates reflect the higher contamination of the German population in metropolitan areas and the lower incidence in sparsely populated areas [3].

In contrast to the general German population afflicted by a high in-hospital case-fatality rate of 17.9 % of hospitalized patients with COVID-19 in the year 2020 [3], our present study showed a very low in-hospital case-fatality rate of 0.23 % in children and young people aged ≤18 years. Our results were in accordance with those of other studies [25,38,40,43]: Götzinger et al. calculated a case-fatality rate of 0.69 % in their multicenter cohort study with participating institutions across 25 European countries during April 2020 [38]. The Center for Disease Control and Prevention (CDC) reported a case-fatality rate of 0.12 % in the United States of America (February 12th to April 2nd, 2020) [40]. In the study of Lu et al., 0.6 % of the children died during hospital stays [22]. Data from the Chinese CDC, including 728 laboratory-confirmed COVID-19 cases from January 16th, 2020, to February 8th, 2020, revealed a case-fatality rate of 0.14 % [43].

Per the literature [22,40], most children with an aggravated course of COVID-19 had preexisting or identified comorbidities. In the present study, heart, liver, and renal failure were identified as important risk factors for an aggravated COVID-19 course (necessity for mechanical ventilation and/or in-hospital fatality) in children. Noticeably in children aged 13–18 years with COVID-19, obesity is an independent risk factor for mechanical ventilation. This is alarming since obesity prevalence in children and adolescents increased substantially during the pandemic in high-income countries [[44], [45], [46]]. It has been recognized that obesity is not only associated with a rise in comorbidities but is also related to an aggravated outcome during COVID-19 infection [3,15,47,48]. While in high-income countries obesity itself is an ongoing pandemic increasing worldwide [47,49,50], in low- and middle-income countries the unprecedented global social and economic crisis triggered by the COVID-19 pandemic is accompanied by an outstanding risk to the nutritional status and survival of young children due to child malnutrition, wasting, driven by declines in household incomes, changes in the availability and affordability of nutritious foods, and interruptions to health, nutrition, and social protection services [51].

In our study, we found that children with COVID-19 had higher rates of heart failure, liver diseases, and kidney diseases compared to those without the infection, especially concerning comorbidities. The proportion of heart failure was 0.7 % of the children with COVID-19 infection in our study, which is more than 3-fold higher than the reported frequency of heart failure (0.2 %) in all hospitalizations of children in the nationwide study of the United States [52]. While the worldwide increase regarding kidney disease is primarily driven by the growing prevalence of diabetes mellitus, arterial hypertension, obesity, and aging [53,54], based on health insurance data of the United States including approximately two million individuals from the pediatric age group (<21 years), the prevalence of children and adolescents with a chronic kidney disease diagnosis code was reported to be 27 per 10,000 (0.27 %) during 2016 [53,55], which is less than one-fourth of the prevalence of acute and/or chronic kidney failure (1.2 %) in our study. Additionally, in the United States, approximately 15,000 children are hospitalized due to liver diseases each year, which constitutes a prevalence of 0.02 % [56]; thus, this calculated prevalence is substantially lower than the 0.9 % of liver of diseases of children ≤18 years infected by COVID-19 investigated in our study. Although the liver is not the primary organ affected by SARS-CoV-2, mild elevation of serum aminotransferases is common in patients with COVID-19 [57]. Notably, the pandemic had a crucial impact on hepatology and other disease-specific services including reduction regarding prevention, testing, treatment, and vaccination services [57]. However, total monthly numbers of cases of liver and renal diseases and heart failure did not increase during the pandemic year 2020 in comparison to the pre-pandemic year 2019 when analyzing all hospitalized children and young people aged ≤18 years in Germany (with and without COVID-19). The primary reasons for this lack of increase in liver, renal, and heart diseases in this young patient group during the pandemic might be most probably driven by the low number of hospitalized children with COVID-19, as well as ambulatory treatment of mild disease forms forced by parents and children's fear of COVID-19 infection and the plan to avoid possible contagion at the emergency departments of the hospitals.

The highest necessity for mechanical ventilation was observed in the youngest patients aged ≤6 years, consistent with previously published studies [38]. The frequency of mechanical ventilation was 3.3 % and thus, similar to the reported rate in the study of Götzinger et al. [38] Unsurprisingly, the necessity of ICU admission and mechanical ventilation were related to substantially increased mortality [3,4].

As the COVID-19 pandemic has unfolded and evolved, reports of children with unusual febrile illnesses and vasculopathy, especially Kawasaki disease, have increased [20,58]. However, data on the incidence and prevalence of vasculopathy in children with COVID-19 are sparse. We detected a vasculopathy frequency of 0.68 % in this patient group. Since vasculopathy related to COVID-19 often co-occurs with other immunological responses [20,58], the rate of multisystem inflammatory syndromes caused by COVID-19 is 0.65 %, similar to that of vasculopathy. Children with multisystem inflammatory syndrome are more prone to developing severe cardiac symptoms that require intensive care [15].

When focusing on the comparison between younger children <10 years and adolescent patients (≥10 to ≤18 years), adolescents suffered more often from acute and chronic lung diseases, but also kidney and liver diseases. This finding support the hypothesis that most adolescent patients are afflicted by a mild or even asymptomatic COVID-19 course not accompanied by a necessity to be hospitalized due to COVID-19 [3,[59], [60], [61], [62], [63], [64], [65]], but comorbidities might trigger hospitalization and a more intense treatment approach [66]. In contrast to these differences between younger children and adolescent patients, no large differences between younger and older adolescent patients were identified. While comorbid profile was similar, the prevalence of pneumonia was more than twofold higher in older compared to younger adolescent patients, whereby no differences regarding mechanical ventilation and in-hospital case-fatality rate were detected. In summary, these results underline the role of existing lung, kidney and liver diseases especially in adolescent COVID-19 patients and highlight that children and adolescents with comorbidities should be prioritized in terms of COVID-19–related health interventions [66].

Grave strain on healthcare systems due to the COVID-19 pandemic worldwide underlines that adequate pediatric services resources have to be allocated and provided to sustain the strain on healthcare systems, guarantee high-quality medicine, and reach the best outcomes for children and young people [38]. In this context, it should not be forgotten that most studies in this context have been conducted in wealthier countries [15,[18], [19], [20], [21], [22], [23], [24], [25], [26], [27], [28], [29]]. However, the pandemic does not stop at national borders, and the experiences and data on the COVID-19 pandemic in poorer countries are of outstanding interest [[29], [30], [31], [32], [33], [34]].

Therefore, reassessing how health systems in poorer countries are handling and managing the pandemic and its consequences should be a focus of further COVID-19 pandemic research. Another important focus should be the influence of COVID-19 and the preventive strategies such as personal hygiene, social distancing, and self-isolation on the humans’ mental well-being [26,28,33,34]. In addition, the influence of the COVID-19 pandemic on childhood obesity warrants careful planning to reduce sedentary lifestyles with childhood obesity as part of the pandemic recovery [45,47,67]. These findings should be considered for future pandemics and should be kept in mind and transferred to other illnesses for optimized medical care. Notably, our study has attracted interest in exacerbating the health complications of conditions that are becoming more common in children (such as diabetes and obesity) [45,47,67]. Obesity is a complex condition related to biological, developmental, environmental, behavioral, and genetic factors [68]. During childhood and adolescence, obesity is most commonly based on inequity in energy balance. Obesity during this early period of life is a significant public health problem and a risk factor for obesity in adolescence and adulthood. The increasing prevalence of obesity in childhood and adolescent obesity is associated with an increase in important life-shortening comorbidities such as diabetes mellitus, arterial hypertension, non-alcoholic fatty liver disease, obstructive sleep apnea syndrome, and dyslipidemia [68]. Therefore, international efforts are needed to prevent obesity and resulting comorbidities, particularly the progression of overweight to obesity and more severe degrees of obesity, and to improve physical activity, exercise, and fitness, as well as overall healthy living, for this age group in general and during future pandemics [47,67,[69], [70], [71], [72], [73], [74]].

The present study has some limitations that merits consideration: Due to the nature of ICD- and OPS-code-based study-analysis of hospitalized patients of the German nationwide-inpatient sample, under-reporting as well as under-coding are possible and data on most concomitant medication or laboratory markers are not available. Also, no follow-up evaluation after discharge from the hospital is available since data are limited to the time-frame of the in-hospital course. Driven by the low adverse event rates CI are wide and precision of the logistic regression results is limited. Since the exact timing and course of comorbidities (i.e. whether they were present on admission or had a new onset during the hospital stay) could not be determined. However, this fact did not reduce the important role of comorbidities as risk factors for adverse outcomes. Finally, we acknowledge that information on the exact cause of death cannot be obtained from the German nationwide inpatient sample.

In conclusion, our findings from an extensive nationwide inpatient dataset in Germany reveal a low in-hospital case fatality rate among individuals aged ≤18 years. Aggravated COVID-19 course is accompanied by underlying comorbidities such as heart, liver and renal failure. Myocarditis, vasculopathy and VTE are rare complications in this patients-group.

## Implications and contribution

This investigation demonstrates that children and adolescents aged ≤18years with COVID-19 infection are fortunately affected by a low case-fatality risk, but heart, liver and renal failure are associated with complicated COVID-19 course. These findings may help to manage the grave strain on health-care systems due to COVID-19 pandemic worldwide.

## Ethical statement

Since our study did not comprise direct access by the investigators to individual patient data but only an access to summarized results provided by the RDC, approval by an ethics committee as well as patients’ informed consent were not required, in accordance with German law.

## Data sharing

All code used in this study is publicly available online. The data used in this study are sensitive due to individual patient-level data and will not be made publicly available. The data is available at the Federal Statistical Office of Germany (Statistisches Bundesamt, DEStatis) (source: RDC of the Federal Statistical Office and the Statistical Offices of the federal states, DRG Statistics 2020, and own calculations).

## Funding

None.

## Declaration of interests

KK, VHS, ISa, VS, OH, FPS report no conflict of interests. SB received lecture/consultant fees from Bayer HealthCare, Concept Medical, BTG Pharmaceuticals, INARI, Boston Scientific, and LeoPharma; institutional grants from Boston Scientific, Bentley, Bayer HealthCare, INARI, Medtronic, Concept Medical, Bard, and Sanofi; and economical support for travel/congress costs from Daiichi Sankyo, BTG Pharmaceuticals, and Bayer HealthCare, outside the submitted work. CEK reports having from Amarin Germany, Amgen GmbH, Bayer Vital, Boehringer Ingelheim, Bristol-Myers Squibb, Daiichi Sankyo, Leo Pharma, MSD Sharp & Dohme, Novartis Pharma, Pfizer Pharma GmbH, Sanofi-Aventis GmbH. SK reports institutional grants and personal lecture/advisory fees from Bayer AG, Daiichi Sankyo, and Boston Scientific; institutional grants from Inari Medical; and personal lecture/advisory fees from MSD and Bristol Myers Squibb/Pfizer. TM reports no conflict of interests. TM is PI of the DZHK (German Center for Cardiovascular Research), Partner Site Rhine-Main, Mainz, Germany. LH received lecture/consultant fees from MSD, Boston Scientific, INARI Medical and Johnson&Johnson, outside the submitted work.
