# Supplementary material for: In-hospital course of children with COVID-19 infection - Results of the German nationwide inpatient sample
Source: Public Health Pract (Oxf). 2025 Jul 1;10:100638. doi: 10.1016/j.puhip.2025.100638 (PMC12281248; doi:10.1016/j.puhip.2025.100638)
Supplement: Multimedia component 1 [file mmc1.docx]

**Supplementary Material**

**In-hospital course of children with COVID-19 infection - Results of the German nationwide inpatient sample**

**Karsten Keller, MD^1,2,3^; Ingo Sagoschen, MD^1^; Volker H. Schmitt, MD^1,4^; Stefano Barco, MD, PhD^2,5^; Sivanathan, Visvakanth, MD^6^; Omar Hahad, MD^1,4^; Frank P. Schmidt, MD^7^; Christine Espinola-Klein, MD^1,2^; Stavros Konstantinides, MD^2,8^; Thomas Münzel, MD^1,4^; Lukas Hobohm, MD^1,2^**

**Affiliations:**

^1^ Department of Cardiology, University Medical Center of the Johannes Gutenberg-University Mainz, Mainz, Germany

^2^ Center for Thrombosis and Hemostasis (CTH), University Medical Center of the Johannes Gutenberg-University Mainz, Mainz, Germany

^3^ Medical Clinic VII, Department of Sports Medicine, University Hospital Heidelberg, Heidelberg, Germany

^4^ German Center for Cardiovascular Research (DZHK), Partner Site Rhine Main, Mainz, Germany

^5^ Department of Angiology, University Hospital Zurich, Zurich, Switzerland

^6^ Department of Gastroenterology, University Medical Center Mainz (Johannes Gutenberg-University Mainz), Mainz, Germany

^7^ Department of Cardiology, Mutterhaus Trier, Germany

^8^ Department of Cardiology, Democritus University of Thrace, Alexandroupolis, Greece

**Corresponding author:**

Karsten Keller, MD, FESC, Department for Cardiology, Cardiology I, University Medical Center Mainz, Johannes Gutenberg-University Mainz Langenbeckstrasse 1, 55131 Mainz Germany. E-mail: Karsten.Keller@unimedizin-mainz.de

**Figure S1:** Temporal monthly trend of total numbers of liver and renal diseases as well as heart failure cases during the pandemic year 2020 and the pre-pandemic year 2019 in all hospitalized children and young people aged ≤18 years in Germany.


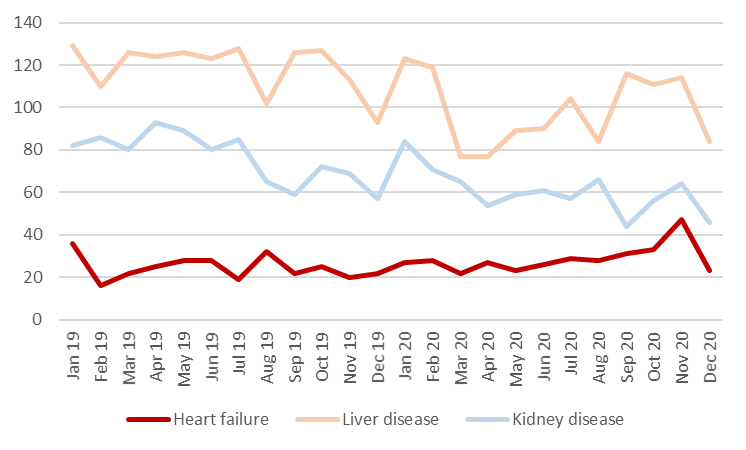


**Table S1:** Patients’ characteristics, medical history, presentation and adverse in-hospital events of the 3,360 hospitalised children with confirmed COVID-19 infection in Germany in the year 2020 stratified for **mechanical ventilation**.

| **Parameters** | **All hospitalized children with COVID-19 aged ≤18 years**  **(n= 3360; 100.0%)** | **Children with COVID-19 without mechanical ventilation**  **(n= 3250; 96.7%)** | **Children with COVID-19 with mechanical ventilation**  **(n= 110; 3.3%)** | **P-value** |
| --- | --- | --- | --- | --- |
| **Age (years)** | 7.0 (0.0-15.0) | 7.0 (0.0-15.0) | 3.5 (0.0-11.3) | **0.002** |
| **Female sex** | 1674 (49.8%) | 1630 (50.2%) | 44 (40.0%) | **0.036** |
| **Length of in-hospital stay** | 2.0 (1.0-4.0) | 2.0 (1.0-4.0) | 10.0 (5.0-19.0) | **<0.001** |
| **Cardiovascular risk factors** | | | |  |
| **Obesity** | 42 (1.3%) | 38 (1.2%) | 4 (3.6%) | 0.047 |
| **Essential arterial hypertension** | 18 (0.5%) | 14 (0.4%) | 4 (3.6%) | **0.002** |
| **Comorbidities** | | | |  |
| **Coronary artery disease** | 3 (0.1%) | 3 (0.1%) | 0 (0%) | 1.000 |
| **Heart failure** | 25 (0.7%) | 14 (0.4%) | 11 (10.0%) | **<0.001** |
| **Atrial fibrillation/flutter** | 3 (0.1%) | * | * | 0.095 |
| **Acute and chronic lung diseases (including bronchial asthma, bronchitis, chronic obstructive pulmonary disease, emphysema, and/or bronchiectasis)** | 67 (2.0%) | 67 (2.1%) | 0 (0%) | 0.172 |
| **Bronchial asthma** | 59 (1.8%) | 59 (1.8%) | 0 (0%) | 0.264 |
| **Acute and/or chronic kidney failure** | 41 (1.2%) | 30 (0.9%) | 11 (10.0%) | **<0.001** |
| **Cancer** | 64 (1.9%) | 59 (1.8%) | 5 (4.5%) | 0.057 |
| **Hepatitis** | 3 (0.1%) | 3 (0.1%) | 0 (0.0%) | 1.000 |
| **Liver disease** | 31 (0.9%) | 28 (0.9%) | 3 (2.7%) | 0.079 |
| **Severe liver disease** | 22 (0.7%) | * | * | 0.161 |
| **Manifestations of COVID-19** | | | |  |
| **Acute bronchitis** | 82 (2.4%) | 79 (2.4%) | 3 (2.7%) | 0.750 |
| **Pneumonia** | 214 (6.4%) | 177 (5.4%) | 37 (33.6%) | **<0.001** |
| **Acute respiratory distress syndrome** | 13 (0.4%) | 6 (0.2%) | 7 (6.4%) | **<0.001** |
| **Vasculopathy** | 23 (0.7%) | * | * | 0.173 |
| **Multisystem Inflammatory syndrome caused by COVID-19** | 22 (0.7%) | 17 (0.5%) | 5 (4.5%) | **0.001** |
| **Recurrent COVID-19 infection after previous COVID-19 infection** | 7 (0.2%) | 7 (0.2%) | 0 (0.0%) | 1.000 |
| **Adverse events during hospitalisation** | | | |  |
| **In-hospital death** | 8 (0.2%) | 5 (0.2%) | 3 (2.7%) | **0.002** |
| **Venous thromboembolism** | 6 (0.2%) | * | * | **0.015** |
| **Acute kidney failure** | 21 (0.6%) | 13 (0.4%) | 8 (7.3%) | **<0.001** |
| **Myocarditis** | 20 (0.6%) | 16 (0.5%) | 4 (3.6%) | **0.004** |
| **Myocardial infarction** | 0 (0%) | 0 (0%) | 0 (0%) | 1.000 |
| **Stroke (ischaemic or haemorrhagic)** | 3 (0.1%) | * | * | **0.003** |
| **Gastro-intestinal bleeding** | 20 (0.6%) | 20 (0.6%) | 0 (0.0%) | 1.000 |
| **Intracerebral bleeding** | 0 (0%) | 0 (0%) | 0 (0%) | 1.000 |
| **Transfusion of blood constituents** | 76 (2.3%) | 52 (1.6%) | 24 (21.8%) | **<0.001** |

* due to confidentiality reasons, the data cannot be shown

**Table S2**: Impact of patient-characteristics and conditions on mechanical ventilation of the 3360 hospitalized children with confirmed COVID-19 infection in Germany in the year 2020 (univariate and multivariate logistic regression model).

|  | **Univariate regression** | | **Multivariate regression*** | |
| --- | --- | --- | --- | --- |
|  | **OR (95% CI)** | **P-value** | **OR (95% CI)** | **P-value** |
| Age (years) | 0.956 (0.929-0.984) | **0.002** | 0.952 (0.923-0.982) | **0.002** |
| Female sex | 0.663 (0.450-0.976) | **0.037** | 0.714 (0.477-1.069) | 0.102 |
| **Cardiovascular risk factors and comorbidities** |  |  |  |  |
| Obesity | 3.190 (1.118-9.099) | **0.003** | 6.119 (2.053-18.235) | **0.001** |
| Arterial hypertension | 8.722 (2.824-26.945) | **<0.001** | 8.134 (2.196-30.126) | **0.002** |
| Diabetes mellitus | 0.639 (0.087-4.676) | 0.659 | 0.781 (0.102-5.970) | 0.812 |
| Heart failure | 25.683 (11.372-57.999) | **<0.001** | 16.957 (6.831-42.094) | **<0.001** |
| Myocarditis | 7.627 (2.507-23.204) | **<0.001** | 1.097 (0.236-5.086) | 0.906 |
| Acute and/or chronic kidney failure | 11.926 (5.810-24.482) | **<0.001** | 9.490 (4.049-22.243) | **<0.001** |
| Acute kidney failure | 19.529 (7.920-48.155) | **<0.001** | 10.865 (3.505-33.676) | **<0.001** |
| Cancer | 2.575 (1.013-6.550) | **0.047** | 2.325 (0.865-6.243) | 0.094 |
| Liver disease | 3.226 (0.966-10.778) | 0.057 | 2.817 (0.775-10.247) | 0.116 |
| Venous thromboembolism | 15.028 (2.723-82.936) | **0.002** | 16.086 (2.165-119.498) | **0.007** |
| **Manifestations of COVID-19** |  |  |  |  |
| Acute bronchitis | 1.125 (0.350-3.622) | 0.843 | 1.281 (0.395-4.161) | 0.680 |
| Pneumonia | 8.800 (5.761-13.441) | **<0.001** | 9.449 (5.885-15.170) | **<0.001** |
| Acute respiratory distress syndrome | 36.744 (12.135-111.265) | **<0.001** | 23.435 (6.070-90.476) | **<0.001** |
| Vasculopathy | 2.847 (0.659-12.298) | 0.161 | 1.143 (0.223-5.863) | 0.873 |
| Multi-segmental Inflammatory syndrome caused by COVID-19 | 9.056 (3.279-25.011) | **<0.001** | 1.636 (0.413-6.481) | 0.483 |

^*^ Adjusted for age, sex, heart failure, acute and chronic lung diseases (including bronchial asthma, bronchitis, chronic obstructive pulmonary disease, emphysema, and/or bronchiectasis), and acute and/or chronic kidney failure.

**Table S3**: Impact of patient-characteristics and conditions on necessity of **mechanical ventilation** in hospitalized children with confirmed COVID-19 infection in Germany in the year 2020 stratified for 6-year cycle (univariate and multivariate logistic regression model).

|  | **Children aged ≤6 years**  **(n= 1640; 48.8%)** | | | | **Children aged 7 to ≤12 years**  **(n= 504; 15.0%)** | | | | **Children aged 13 to ≤18 years**  **(n= 1216; 36.2%)** | | | |
| --- | --- | --- | --- | --- | --- | --- | --- | --- | --- | --- | --- | --- |
|  | **Univariate regression** | | **Multivariate regression*** | | **Univariate regression** | | **Multivariate regression*** | | **Univariate regression** | | **Multivariate regression*** | |
|  | **OR (95% CI)** | **P-value** | **OR (95% CI)** | **P-value** | **OR (95% CI)** | **P-value** | **OR (95% CI)** | **P-value** | **OR (95% CI)** | **P-value** | **OR (95% CI)** | **P-value** |
| Female sex | 0.602 (0.360-1.009) | 0.054 | 0.537 (0.312-0.926) | **0.025** | 2.078 (0.728-5.929) | 0.172 | 2.004 (0.677-5.934) | 0.209 | 0.612 (0.281-1.333) | 0.217 | 0.720 (0.319-1.627) | 0.430 |
| **Cardiovascular risk factors and comorbidities** |  |  |  |  |  |  |  |  |  |  |  |  |
| Obesity | - | - | - | - | 4.918 (0.566-42.717) | 0.149 | 5.891 (0.659-52.665) | 0.113 | 5.043 (1.436-17.719) | **0.012** | 6.758 (1.876-24.353) | **0.003** |
| Diabetes mellitus | - | - | - | - | 3.810 (0.451-32.160) | 0.219 | 4.841 (0.555-42.230) | 0.154 | - | - | - | - |
| Heart failure | 24.841 (7.793-79.184) | **<0.001** | 19.091 (5.407-67.406) | **<0.001** | 11.571 (1.132-118.302) | **0.039** | 3.653 (0.235-56.862) | 0.355 | 43.091 (10.832-171.413) | **<0.001** | 28.966 (6.718-124.890) | **<0.001** |
| Myocarditis | 5.761 (0.635-52.239) | 0.120 | 1.487 (0.111-19.968) | 0.765 | 11.571 (1.132-118.302) | **0.039** | 2.352 (0.111-49.754) | 0.583 | 10.935 (2.242-53.333) | **0.003** | 0.578 (0.046-7.290) | 0.672 |
| Acute and/or chronic kidney failure | 29.829 (8.866-100.355) | **<0.001** | 23.786 (6.405-88.326) | **<0.001** | 14.892 (2.641-83.992) | **0.002** | 8.643 (1.222-61.129) | **0.031** | 7.630 (2.118-27.492) | **0.002** | 4.458 (0.985-20.188) | 0.052 |
| Cancer | 1.707 (0.398-7.328) | 0.472 | 1.501 (0.310-7.270) | 0.614 | 4.862 (1.006-23.483) | **0.049** | 6.380 (1.263-32.229) | **0.025** | 2.760 (0.353-21.554) | **0.021** | 2.107 (0.242-18.343) | 0.500 |
| Liver disease | 11.694 (2.105-64.973) | **0.005** | 13.192 (2.333-74.585) | **0.004** | - | **-** | - | **-** | 2.935 (0.375-22.999) | 0.305 | 2.091 (0.228-19.160) | 0.514 |
| Venous thromboembolism | 11.537 (1.033-128.797) | **0.047** | 16.106 (1.394-186.117) | **0.026** | - | **-** | - | **-** | 23.760 (2.086-270.675) | **0.011** | 9.832 (0.228-424.837) | 0.234 |
| **Manifestations of COVID-19** |  |  |  |  |  |  |  |  |  |  |  |  |
| Acute bronchitis | 1.204 (0.285-5.097) | 0.801 | 1.309 (0.307-5.585) | 0.716 | - | - | - | - | 1.360 (0.179-10.331) | 0.766 | 1.602 (0.207-12.384) | 0.651 |
| Pneumonia | 8.646 (4.639-16.114) | **<0.001** | 8.155 (4.209-15.803) | **<0.001** | 16.491 (5.326-51.063) | **<0.001** | 16.394 (4.819-55.774) | **<0.001** | 14.091 (6.310-31.467) | **<0.001** | 12.790 (5.430-30.127) | **<0.001** |
| Acute respiratory distress syndrome | 71.364 (7.325-695.284 | **<0.001** | 70.115 (6.201-792.737) | **0.001** | 75.077 (6.396-881.256) | **0.001** | 48.694 (2.088-1135.842) | **0.016** | 24.708 (4.316-141.452) | **<0.001** | 11.067 (1.213-100.998) | **0.033** |
| Vasculopathy | 1.762 (0.227-13.669) | 0.588 | 0.580 (0.051-6.573) | 0.660 | - | - | - | - | 15.827 (1.591-157.475) | **0.018** | 4.182 (0.210-83.392) | 0.349 |
| Multi-segmental Inflammatory syndrome caused by COVID-19 | 5.181 (1.098-24.447) | **0.038** | 0.756 (0.081-7.033) | 0.806 | - | - | - | - | 30.913 (6.969-137.115) | **<0.001** | 3.890 (0.429-35.296) | 0.227 |

^* Adjusted for sex, heart failure, acute and chronic lung diseases (including bronchial asthma, bronchits, chronic obstructive pulmonary disease, emphysema, and/or bronchiectasis), and acute and/or chronic kidney failure.^

**Table S4**: Impact of patient-characteristics and conditions on in-hospital **case-fatality** in hospitalized children with confirmed COVID-19 infection in Germany in the year 2020 stratified for 6-year cycle (univariate logistic regression model).

|  | **Children aged ≤6 years (n= 1640; 48.8%)** | | **Children aged 7 to ≤12 years (n= 504; 15.0%)** | | **Children aged 13 to ≤18 years (n= 1216; 36.2%)** | |
| --- | --- | --- | --- | --- | --- | --- |
|  | **Univariate regression** | | **Univariate regression** | | **Univariate regression** | |
|  | **OR (95% CI)** | **P-value** | **OR (95% CI)** | **P-value** | **OR (95% CI)** | **P-value** |
| Female sex | 1.314 (0.082-21.037) | 0.847 | 2.726 (0.246-30.265) | 0.414 | **-** | **-** |
| **Cardiovascular risk factors and comorbidities** |  |  |  |  |  |  |
| Heart failure | 147.909 (8.689-2517.742) | **0.001** | **-** | **-** | **-** | **-** |
| Myocarditis | - | **-** | 83.000 (5.833-1180.977) | **0.001** | **-** | **-** |
| Acute and/or chronic kidney failure | 162.800 (9.505-2788.323) | **<0.001** | 41.250 (3.280-518.784) | **0.004** | 27.068 (2.366-309.691) | **0.008** |
| Acute kidney failure | 408.500 (21.588-7729.796) | **<0.001** | 62.125 (4.641-831.663) | **0.002** | 60.150 (5.038-718.141) | **0.001** |
| Cancer | - | **-** | **-** | **-** | 35.176 (3.042-406.712) | **0.004** |
| **Manifestations of COVID-19** |  |  |  |  |  |  |
| Pneumonia | 23.088 (1.429-373.061) | **0.027** | 41.565 (3.635-475.283) | **0.003** | **-** | **-** |
| Acute respiratory distress syndrome | - | **-** | 124.750 (7.792-1997.212) | **0.001** | 120.800 (9.375-1556.568) | **<0.001** |
| **Treatment** |  |  |  |  |  |  |
| Mechanical ventilation | 23.088 (1.429-373.061) | **0.027** | 17.393 (1.488-203.285) | **0.023** | 23.760 (2.086-270.675) | **0.011** |
